# Core Gene Set As the Basis of Multilocus Sequence Analysis of the Subclass Actinobacteridae

**DOI:** 10.1371/journal.pone.0014792

**Published:** 2011-03-31

**Authors:** Toïdi Adékambi, Ray W. Butler, Finnian Hanrahan, Arthur L. Delcher, Michel Drancourt, Thomas M. Shinnick

**Affiliations:** 1 Mycobacteriology Laboratory Branch, Division of Tuberculosis Elimination, National Center for HIV/AIDS, Viral Hepatitis, STD, and TB Prevention, Centers for Disease Control and Prevention, Atlanta, Georgia, United States of America; 2 Center for Bioinformatics and Computational Biology, University of Maryland, College Park, Maryland, United States of America; 3 Unité de Recherche sur les Maladies Infectieuses et Tropicales Emergentes, CNRS UMR 6236, IRD 198, IFR 48, Faculté de Médecine, Université de la Méditerranée, Marseille, France; University of Liverpool, United Kingdom

## Abstract

Comparative genomic sequencing is shedding new light on bacterial identification, taxonomy and phylogeny. An in silico assessment of a core gene set necessary for cellular functioning was made to determine a consensus set of genes that would be useful for the identification, taxonomy and phylogeny of the species belonging to the subclass Actinobacteridae which contained two orders *Actinomycetales* and *Bifidobacteriales*. The subclass Actinobacteridae comprised about 85% of the actinobacteria families. The following recommended criteria were used to establish a comprehensive gene set; the gene should (i) be long enough to contain phylogenetically useful information, (ii) not be subject to horizontal gene transfer, (iii) be a single copy (iv) have at least two regions sufficiently conserved that allow the design of amplification and sequencing primers and (v) predict whole-genome relationships. We applied these constraints to 50 different Actinobacteridae genomes and made 1,224 pairwise comparisons of the genome conserved regions and gene fragments obtained by using Sequence VARiability Analysis Program (SVARAP), which allow designing the primers. Following a comparative statistical modeling phase, 3 gene fragments were selected, *ych*F, *rpo*B, and *sec*Y with R^2^>0.85. Selected sets of broad range primers were tested from the 3 gene fragments and were demonstrated to be useful for amplification and sequencing of 25 species belonging to 9 genera of Actinobacteridae. The intraspecies similarities were 96.3–100% for *ych*F, 97.8–100% for *rpo*B and 96.9–100% for *sec*Y among 73 strains belonging to 15 species of the subclass Actinobacteridae compare to 99.4–100% for 16S rRNA. The phylogenetic topology obtained from the combined datasets *ych*F+*rpo*B+*sec*Y was globally similar to that inferred from the 16S rRNA but with higher confidence. It was concluded that multi-locus sequence analysis using core gene set might represent the first consensus and valid approach for investigating the bacterial identification, phylogeny and taxonomy.

## Introduction

Beyond the standard DNA-DNA hybridization value, the ad-hoc committee concerned with the revaluation of taxonomy of the species definition in bacteriology proposed using a small set of five housekeeping genes for quantitative evaluation of taxonomic relatedness to achieve an adequately informative level of phylogeny data and issued a call for the detection of such genes [1]. Previous studies have confirmed that sequences of housekeeping genes accurately predict genome relatedness and can be used for species-level identification [2], [3].

Several housekeeping genes have been used for bacterial phylogeny and identification [4]–[6] but no consensus has been made regarding an optimal selection of genes. Also, some previously recommended genes were absent in some species or isolates of the same genus or could not be amplified [5], [7]. For example, 695 protein coding sequences were present in *Mycobacterium marinum* but not in *Mycobacterium ulcerans* or *Mycobacterium tuberculosis* [8]. Admittedly, these studies were empirical and did not take advantage of currently available genome sequences.

Comparative genome sequence analysis may also aid in the reasonable selection of candidate genes with defined characteristics. It has been reported that suitable genes should fulfill the following conditions; (i) the genes must be ubiquitous with orthologous sequences in all cellular life as is the case of 16S rRNA gene; (ii) the genes must be present in single copy among genomes, without close paralogues that could confuse analysis; (iii) the individual genes must be long enough (>900 bases) to contain sufficient information; (iv) the genes should not be prone to horizontal gene transfer (HGT) or recombination; (v) closely linked genes should also be avoided; (vi) the genes should contain at least two highly conserved regions to allow the design of appropriate amplification and sequencing primers and (vii) the sequence must predict whole-genome relationships with acceptable precision [3], [9]–[11].

In this study, we applied these conditions to the analysis of the subclass Actinobacteridae using genome computational methods to deduce a small set of useful genes [12]. The development of a universal approach for housekeeping genes for classification and identification (as is the case of 16S rRNA gene) may present difficulties because of the saturation of the third codon position over a long evolutionary timescale [13]. So, for the subclass Actinobacteridae, we applied these conditions to the 63 core genes originally suggested by Koonin [14] who compared 100 sequenced genomes belonging to the all cellular life (Bacteria, Archaea and Eukaryotes). A common set of genes was previously defined as the smallest possible group of genes that would be sufficient to sustain a functioning cellular life form under the most favorable conditions imaginable, that is, in the presence of a full complement of essential nutrients and in the absence of environmental stress [15], [16]. For many important pathogens, the genes common to all strains within a species [known as the core genome] are a minority component of the entire gene pool for that species (the pan-genome) [17].

The subclass Actinobacteridae contained two orders *Actinomycetales* and *Bifidobacteriales* and comprised about 85% of the actinobacteria families [18]. They are Gram-positive bacteria with a high G+C content in their DNA ranging from 51% in some *Corynebacterium species* to more than 70% in *Streptomyces* and *Frankia* species; 43 families and about 200 genera are recognized. This lineage comprises a wide range of morphologically diverse organisms with different phenotypic characteristics; from coccoid (e. g. *Micrococcus*) or rod-coccoid (e.g. *Arthrobacter*) to fragmenting hyphal forms (e.g. *Nocardia* spp.) or permanent and highly differentiated branched mycelium (e.g. *Streptomyces*) [18]–[20]. The actinobacteria have a common ancestry [18], [21], [22] and share conserved indels in protein sequences [23] and 23S rRNA [24] that are characteristics of this phylum. A recent comprehensive analysis of four actinobacterial genomes identified 233 proteins with unknown functions that were unique for this cluster of genomes and do not have homologues in any other currently available bacterial genome [25]. Some genera of the subclass Actinobacteridae are under taxonomic reevaluation [26]–[33]. Unique biochemical characteristics shared by all subclass members have not been demonstrated. Many members of the subclass stain acid-alcohol-fast but may not represent known families [34]. Some Mycobacteriology reference laboratories also identify *Nocardia* and *Rhodococcus* species. Therefore, it is noteworthy to find consensus genes, particularly those with robust, broad range primers useful for the taxonomic, phylogenetic, and identification analysis of this subclass.

Therefore, we systemically analyzed with computational tools, genes belonging to the so-called core gene set defined based on genome examination of *Actinobacteridae* in order to (i) determine a standard set of genes for use in all phylogenetic levels and (ii) complement and extend the utility of the 16S rRNA gene in the identification and classification of bacteria. Among the core set genes, we attempted to detect genes useful for the first line identification and taxonomic relationship analysis of the subclass Actinobacteridae. In this study, we proposed a core gene set as consensus genes for multi-locus sequence analysis (MLSA) for the subclass Actinobacteridae.

## Results

### Genes selection

**Figure 1:**
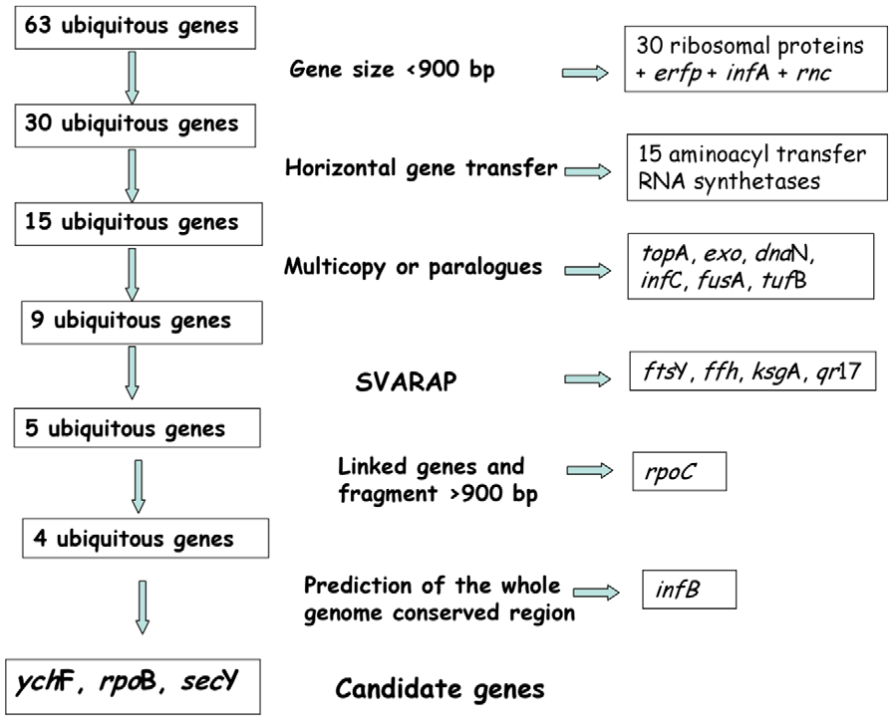
Strategy to establish a comprehensive gene set useful for MLSA following the literature recommended criteria.

For the 63 ubiquitous genes identified by Koonin [14], we used the algorithm described in **Figure 1**. More than half were eliminated because they had sequence length less than 900 bp (312–840 bp). Most of these genes belong to the family of ribosomal proteins (48%; 30/63 genes). Also, the aminoacyl-transfer-RNA synthetase genes (24%; 15/63 genes) presented evidence of HGT that limited their usefulness for bacterial taxonomy and identification [35]–[37]. Furthermore, it has been shown that ribosomal proteins and tRNA synthetases are not appropriate for use in phylogenetic analyses [38] due to their small size and HGT. Therefore, these genes were removed from the study. BLAST searches were used to eliminate candidates that had multiple copies or close paralogues in the genome (**Figure 1**). After application of these criteria, only 9 candidate genes (15%) remained for further analysis of the nucleotides with sequence alignments (**Figure 1**). These candidate genes were categorized in three classes; transcription (*rpo*B, *rpo*C), translation (*inf*B, *ych*F, *ksg*A, *qr*17) and secretion (*sec*Y, *ffh*, *fts*Y) pathways. Among the candidate genes, 2 pairs were linked (*rpo*B-*rpo*C and *fts*Y-*ffh*).

### Primer selection

**Figure 2:**
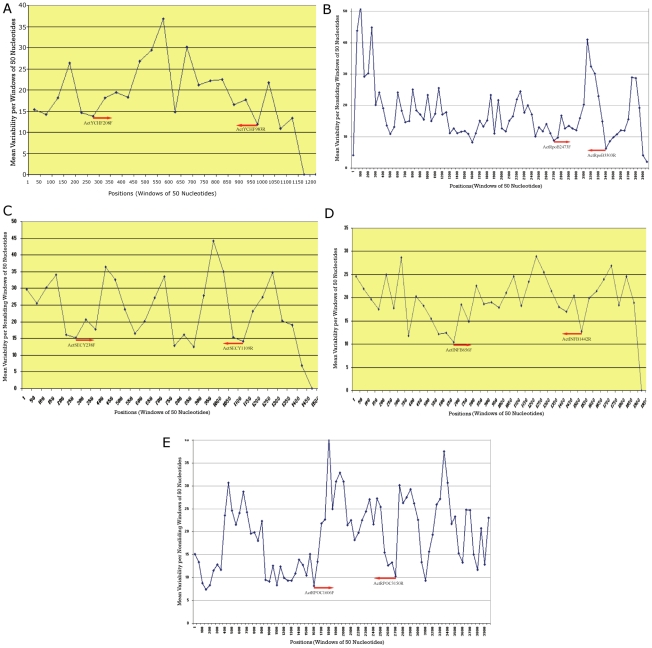
Mean variability for successive windows of 50 nucleotide positions using SVARAP. (A) *ych*F gene sequence,(B) *rpo*B gene sequence (C), *sec*Y gene sequence (D), *inf*B gene sequence and (E) *rpo*C gene sequence The arrows correspond to the forward and reverse amplification and sequencing primers.

After applying Sequence VARiability Analysis Program (SVARAP) to the 9 candidate genes [39], [40], two genes, *ksg*A and *qr*17, contained only a single well-conserved region to design a low-degeneracy primer [data not shown]. Primers were designed for 6 functionally diverse genes in the conserved regions flanking the hypervariable regions. The mean variability of the highly conserved regions where the primers were selected was <15% (**Figure 2A, 2B, 2C, 2D and 2E**). For several of the selected genes (*rpo*B, *rpo*C, *inf*B), there were numerous potential primer binding sites conserved across the studied genera. But our constraint was to choose a hypervariable region flanked by conserved regions which potentially had enough information to distinguish different Actinobacteridae species and strains. Also, an accommodating fragment size from 600–900 bp was needed in other to be sequenced directly in both directions with the PCR primers.

### Primers tested

The primers were tested with 26 species belonging to 10 Actinobacteria genera. Four primer combinations of the *ffh* and two primer combinations of *fts*Y gene produced specific, non-specific and negative amplification among all tested species. Therefore, these genes were not considered further.

One set of primers for each of the 5 genes (*rpo*B, *rpo*C *inf*B, *ych*F and *sec*Y) was identified with the criteria enumerated in materials and methods (**Figure 1**). There were 5 primer pairs which bind across all these bacterial taxa. For 4 of the 5 genes (exception *rpo*C gene), their fragments could be sequenced directly in both directions with the PCR primers by using the ABI prism 3130*xl* instrument. Among the 50 genomes understudy, the sizes ranged from 700–718 bp for *ych*F (∼65% coverage), 745–769 bp for *rpo*B (∼22% coverage), 766–793 bp for *sec*Y (∼60% coverage), and 733–745 bp for *inf*B (∼27% coverage).

Ultimately, we identified (see prediction of whole genome conserved region analysis) one combination of primers for each of the 3 genes (*ych*F, *rpo*B and *sec*Y) that produced a single amplicon for each of the phylogenetically diverse Actinobacteridae species screened (**Table 1**). Their size (according to *M. tuberculosis* numbering Genbank accession number CP000611) and annealing temperature were summarized in **Table 2**. The *sec*Y primers cannot amplify *Atopobium rimae*, an actinobacteria that is not belonging to the subclass Actinobacteridae (**Table 1**). In addition, in-silico analysis on the 12 species that belonged to the phylum Actinobacteria but not to the subclass Actinobacteridae showed in some cases restricted binding sites for either the *sec*Y or *ych*F primers but not for the *rpo*B primers. Therefore, the analysis in the present work is restricted on the subclass Actinobacteridae. The intraspecies similarities were 96.3–100% for *ych*F, 97.8–100% for *rpo*B and 96.9–100% for *sec*Y among 73 strains belonging to 15 species of Actinobacteridae compare to 99.4–100% for 16S rRNA (**Table 3**). The list of the primers specific for the common set of genes described above can be used for the amplification and sequencing of these same genes in other genera in the subclass Actinobacteridae.

**Table 1:** Testing amplification with broad range primers on some representative genera of the subclass Actinobacteridae. (+) PCR positif.

	16S rRNA	*ych*F	*rpo*B	*sec*Y
*Nocardia asteriodes*	+	+	+	+
*Nocardia brasiliense*	+	+	+	+
*Nocardia otitidiscaviarum*	+	+	+	+
*Actinomadura madurae*	+	+	+	+
*Actinomadura pelletieri*	+	+	+	+
*Tsukamurella aurantiacutis*	+	+	+	+
*Tsukamurella paurometabolum*	+	+	+	+
*Corynebacterium jeikeium*	+	+	+	+
*Corynebacterium xerosis*	+	+	+	+
*Corynebacterium pseudotuberculosis*	+	+	+	+
*Gordonia bronchialis*	+	+	+	+
*Gordonia sputi*	+	+	+	+
*Gordonia terrae*	+	+	+	+
*Rhodococcus equi*	+	+	+	+
*Rhodococcus erythropolis*	+	+	+	+
*Streptomyces somaliensis*	+	+	+	+
*Mycobacterium avium*	+	+	+	+
*Mycobacterium kansasii*	+	+	+	+
*Mycobacterium fortuitum*	+	+	+	+
*Mycobacterium lentiflavum*	+	+	+	+
*Mycobacterium goodii*	+	+	+	+
*Mycobacterium salmoniphilum*	+	+	+	+
*Mycobacterium simiae*	+	+	+	+
*Mycobacterium tuberculosis*	+	+	+	+
*Bifidobacterium longum*	+	+	+	+
*Atopobium rimae*	+	+	+	−

**Table 2:** Broad range primer sequences for amplification and sequencing of the species of the subclass Actinobacteridae.

Genes name	Sequence of the primers	Excepted sequence size (*M. tuberculosis* numbering)	Hybridization Temperature
*rpo*B	ActRpoB2473F: GGHAAGGTSACSCCNAAGGG ActRpoB3303R: GAANCGCTGDCCRCCGAACTG	754 bp	60°C
*sec*Y	ActSECY238F: GGBRTBATGCCSTACATYAC ActSECY1109R: AANCCRCCRWACTKCTTCAT	787 bp	52°C
*ych*F	ActYCHF208F: TTYGTBGAYATCGCVGG ActYCHF983R: ACGAYYTCVGCYTTGATGAA	703 bp	52°C

R = A or G; Y = C or T; K = G or T; S = G or C; W = A or T; B = C, G, or T; D = A, G, or T; H = A, C, or T; V = A, C, or G; N = A, C, G, or T.

**Table 3:** Core gene set intraspecies similarities among species of the subclass Actinobacteridae.

Species	Number of isolates	16S rRNA similarity (%)	*ych*F similarity (%)	*rpo*B similarity (%)	*sec*Y similarity (%)
***Mycobacterium abscessus*** ** sensu stricto^(a)^**	**20**	**100**	**99.6–100**	**99.6–100**	**99.7–100**
*Mycobacterium avium*	3	100	99.1–99.9	99.7	99.9–100
*Mycobacterium leprae*	2	100	100	100	100
*Mycobacterium bovis*	3	100	100	100	100
*Mycobacterium tuberculosis*	20	100	100	100	100
*Mycobacterium monacense*	3	100	99.0–100	100	99.6–100
*Rhodococcus erythropolis*	2	100	96.9	99.3	96.3
*Corynebacterium glucuronolyticum*	2	100	100	99.7	100
*Corynebacterium matruchotii*	2	99.4	99.9	98.8	99.2
*Streptomyces roseosporus*	2	100	100	99.9	100
*Propionibacterium acnes*	2	99.9–100	100	99.9	100
*Bifidobacterium adolescentis*	2	98.7–99.9	97.6	99.2	99.2
*Bifidobacterium animalis*	4	99.8–100	100	100	100
*Bifidobacterium longum*	4	99.9–100	98.3–100	97.8–99.9	98.1–100
*Tropheryma whipplei*	2	100	99.8	99.7	100

(a) The intraspecies similarities were performed experimentally on 20 clinical isolates of *Mycobacterium abscessus.*

### Candidate gene fragment sequences and predicting whole genome conserved region relationships

**Figure 3:**
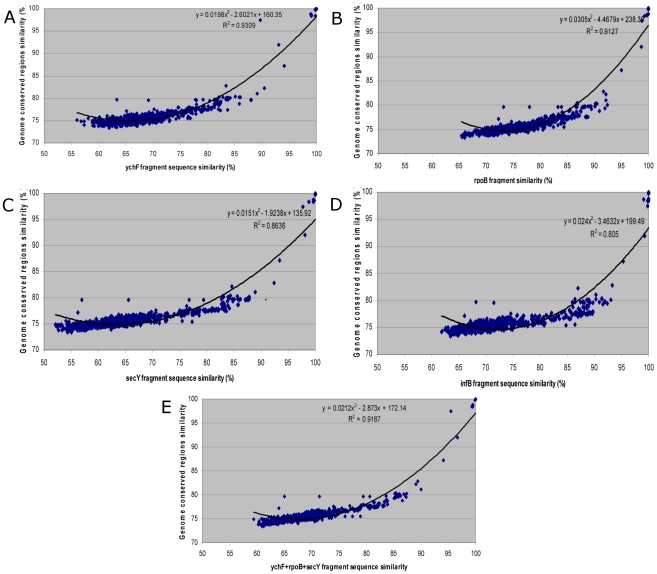
Scatter plot of the relationship between genome conserved similarity and gene sequence fragment similarity. (A) *ych*F fragment, (B) *rpo*B fragment, (C) *sec*Y fragment, (D), *inf*B fragment and (E) *ych*F+*rpo*B+*sec*Y fragment.

Because MLSA is based on the sequences of housekeeping gene fragments, it remained to be determined which of the candidate gene fragment sequences best satisfied the final criterion by predicting whole genome conserved region relationships. The scatter plot of the gene fragment similarities and the whole genome conserved region similarities (n = 1,224 pairwise comparisons) are shown in **Figure 3A, 3B, 3C and 3D**. A least-squares quadratic regression was computed for each gene, giving the following formulae for the whole genome conserved region relatedness (GCR) for two actinobacterial strains as a function of gene-fragment sequence similarity (SS):
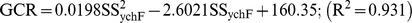


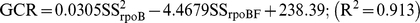


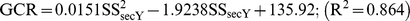


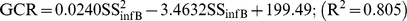



The best three gene fragments were *ych*F, *rpo*B and *sec*Y, all with R^2^ above 0.85 while *inf*B had a somewhat lower value. Interestingly, combining of the three top gene fragments produced a prediction model with a high R^2^ value of 0.919 and formula: GCR = 0.0212SS^2^_ychFrpoBsecY_−2.873SS_ychFrpoBsecY_ +172.14 (**Figure 3E**). Among the 50 genomes studied, the sizes ranged from 2,220–2,274 bp for *ych*F+*rpo*B+*sec*Y.

### Phylogenetic analysis

**Figure 4:**
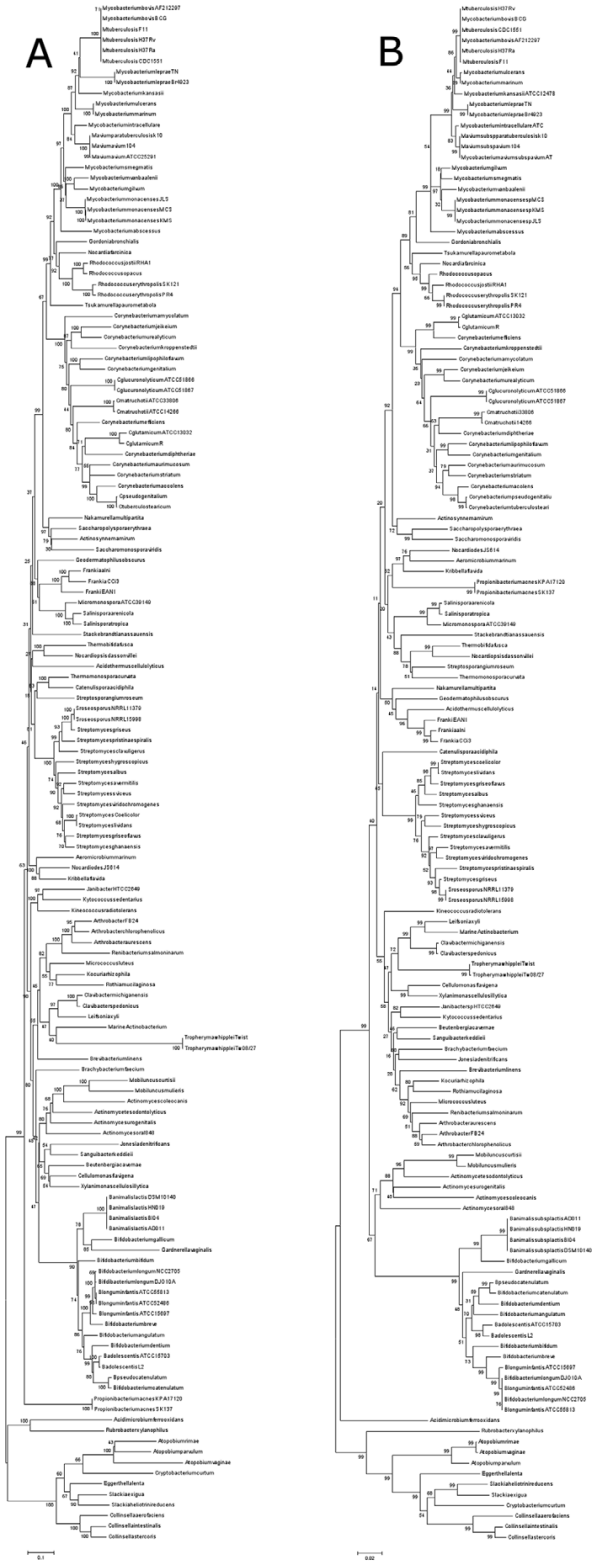
Phylogenetic tree of actinobacterial species using the neighbour-joining method with Kimura's two parameter distance correction. (A) *ych*F+*rpo*B+*sec*Y fragment, (B) 16S rRNA gene. The support of each branch, as determined from 1000 bootstrap samples, is indicated by the value at each node (as a percentage). Bar represent difference in nucleotide sequences.

In addition to the 50 species understudy, 83 species of the subclass Actinobacteridae that the genome sequences are in the pipeline are included in the phylogenetic analysis. Species (n = 12) that belonged to the phylum Actinobacteria but not to the subclass Actinobacteridae are used as outgroup. The phylogenetic trees constructed with different fragment of genes were shown to have moderate heterogeneity in term of topological histories (data not shown). However, the concatenation of these 3 gene fragments delivered high confidence in phylogenetic tree as shown in the **Figure 4A** by using neighour joining method. The percentage of bootstrap values greater than 65% at each node was higher for *ych*F+*rpo*B+*sec*Y (81%) compare to 16S rRNA (68%) (p = 0.01 by chi-square test). A bootstrap value >67% in the concatenated tree supported the fork separating *Mycobacterium Corynebacterium, Streptomyces*, and *Bifidobacterium* genera from the other recognized genera (**Figure 4A**). The phylogenetic organizations obtained from combined datasets were globally similar to that inferred from the 16S rRNA (**Figure 4B**) but with higher confidence indicating that *ych*F+*rpo*B+*sec*Y appeared to be useful tool in addition to the 16S rRNA gene for the investigation of evolutionary relationships among the species of the subclass Actinobacteridae.

## Discussion

Analysis of the 16S rRNA gene sequences has served as the standard to assess Actinobacteria diversity in nature and to classify Actinobacteria species [21]. Recently, new specific 16S rRNA gene primers were designed and an Actinobacteria Amplification Resource (http://microbe2.ncl.ac.uk/MMB/AAR.htm) site was constructed to provide a visual guide to aid in the amplification of actinobacterial 16S rRNA gene in marine and terrestrial environment [41]. The appeal of these molecules lies in their ubiquitous distribution and relatively slow rate of evolution, which enables comparison among divergent Actinobacteria species. Several authors have noted shortcomings in using 16S rRNA gene sequences for assessing Actinobacteria diversity and for phylogenetic analysis. The lack of informative characters and a slow evolution rate complicates both the differentiation of closely related strains of bacteria as well as the resolution of an evolutionary tree [42]. 16S rRNA is a multiple copy gene and may be present in 1–6 copies in the subclass Actinobacteridae (www.genome.jp) with 99.4–100% similarity (Table 3). Some of these multiple copies of 16S rRNA gene exhibit different sequences [4], [43], [44]. In this situation, direct sequencing is not suitable for isolate identification because of discrepant results are produced by the different sequences. In the vast majority of bacterial genomes, the divergence between 16S rRNA gene sequence copies is <1% [45]. Also, the influence of intragenomic heterogeneity displayed by the 16S rRNA gene on bacterial phylogeny was assessed.

Furthermore, despite the perceived reliability of the 16S rRNA gene sequence as a phylogenetic marker, it is known that any single measure of sequence similarity is subject both to simple stochastic variation and to the influence of recombination or HGT [35], [46]. Also, examples of HGT of the 16S rRNA in nature have been reported based on patterns of the 16S rRNA gene sequence heterogeneity, but these are limited to relatively closely related organisms, including certain *Actinomycetes* [44], [47], [48].

MLSA has been proposed as an alternative to 16S rRNA for some genera of the subclass Actinobacteridae such as *Mycobacterium* [4]–[6], *Bifidobacterium* [49], *Microbacterium* [50], [51], and *Streptomyces* [52]. However, there was no consensus regarding the choice of genes to be used for MLSA amongst these genera and choices have remained empirical. The increasing genomic database was examined to define a rational post-genomic study of a common set of genes that may be useful for the MLSA classification, phylogeny and identification of the species belonging to the subclass Actinobacteridae.

The present study represented a first attempt in the development of a systematic measured approach for proposal of a core set of genes for MLSA useful for the subclass Actinobacteridae. The main objective was the selection of housekeeping genes as candidates that belong to the common set of genes and fulfilled the criteria noted before. The availability of 50 complete genomes of the subclass Actinobacteridae provided the stimulus for selecting the candidate loci. The hypothesis that gene fragment sequence can predict genome conserved region accurately is supported strongly in this study. The 3 gene fragments (*ych*F, *rpo*B and *sec*Y) selected appeared to be stable and evolved slowly. The phylogenetic tree derived from the concatenation of these 3 fragments is more robust than that derived from the 16S rRNA (**Figure 4A and 4B**). The 3 loci selected were found to be suitable for MLSA as they amplified and could be sequenced in the species of the subclass Actinobacteridae studied. Also, certain loci were linked as in the case of *rpo*B and *rpo*C (*rpo*B always preceding *rpo*C). According to the large *rpo*B database [53] and because the *rpo*C amplicon (>1300 bp) was sequenced totally only with additional sequence primers (**Figure 2E**), we proposed to incorporate *rpo*B rather than *rpo*C in the MLSA. To our knowledge, these 3 loci have not been incorporated in the same MLSA studies and this represents the first time that *ych*F gene has been proposed for bacterial taxonomy, phylogeny and identification. Furthermore, the 3 fragments distinguish more the strains of a single species than the 16S rRNA (Table 3). Although there are no validated cut-off values to delineate species of the subclass Actinobacteridae, we observed that similarity values of <96.3% for *ych*F, <97.8% for *rpo*B and <96.9% for *sec*Y effectively delineated the currently recognized species in the subclass Actinobacteridae. Similar threshold (97.7%) has been suggested for the *rpo*B by analysing the complete sequence [53]. However, *M. marinum* and *M. ulcerans*; *Rhodococcus jostii* and *Rhodococcus opacus*; *Corynebacterium pseudogenitalium* and *Corynebacterium tuberculostearium*; *Streptomyces roseosporus* and *Streptomyces griseus*; *Streptomyces coelicolor* and *Streptomyces lividans; Bifidobacterium catenulatum* and *Bifidobacterium pseudocatenulatum* seem to belong to the same species.

Contrary to the slow evolution rate of the 16S rRNA gene, these 3 genes belonging to the essential gene set tend to be highly evolutionarily conserved, in terms of both the rate of sequence evolution [54] and particularly, in terms of wide phyletic spread [54]–[56]. It was suggested by Zeigler [3] that less than five genes might be sufficient to equal or surpass the power of DNA-DNA hybridizations and could predict overall phylogenetic relatedness with high precision. Recently, it was described by Edwards [57] that it was not so much the multiplicity of genes that was deemed responsible for the success of combining information via concatenation, but rather the multiplicity of characters or sites. We also demonstrated that *rpo*B sequence similarity was significantly correlated with DNA-DNA hybridization among two bacterial species [58] and average nucleotide identity [59].

Despite the concerns with MLSA due to the difficulties in choosing genes to be compared, the information derived from the common set of genes presented here can complement and extend the utility of the 16S rRNA sequences for resolving issues pertaining to the genetics and evolution of bacterial genomes. Based on the consensus view introduced here, this common set of genes described may serve as a convenient starting point in the logical development of MLSA for other bacterial species and may be useful in construction of a supertree [60]. In this study, we systematically selected gene fragments of *ych*F, *rpo*B and *sec*Y as suitable representative candidates to achieve the goal of creating and generating a robust and highly discriminatory supertree which infers phylogeny among members of Actinobacteria species. Moreover, these 3 fragments of genes could potentially reflect the evolution of the whole genome because they are spaced well apart on the genome and their tree heterogeneity is moderate.

Using a common set of genes for MLSA would represent an easier way to standardize the identification and phylogenetical relationships of known and unknown species across the subclass Actinobacteridae. Admittedly, further studies will be necessary needed to assess the intraspecies and the interspecies variability of isolates and reference strains in the different genera to improve some guidelines for the use of the common set of genes [40]. MLSA would also favour the creation of sequence databases for comparative purposes and would allow taxonomists to compare new taxa at a remote location via the internet. The exchange of reference strains between laboratories could be reduced and this approach could aid the reorganization of the species of the subclass Actinobacteridae, which would be important for misclassified species and unnamed taxa. Finally, the approach described above may have universal application but should be tested with other bacterial subclass. The primer sets will likely have to be adapted for each subclass or bacterial group.

In summary a set of broad range primers were developed that targeted housekeeping genes distributed in the subclass Actinobacteridae. From the data presented, we concluded that MLSA using the common set of genes *ych*F, *rpo*B, and *sec*Y represented a valid approach for investigating the identification, phylogeny and taxonomy of Actinobacteria genera and may represent an alternative approach to DNA-DNA hybridization.

## Materials and Methods

### Strains and an *in silico* core gene set databases

Genomes of 50 different species of the subclass Actinobacteridae including multiple strains of the same species have been sequenced to completion (**Table S1**) [61]. An *in silico* core gene set database was constructed from these genomes based on the 63 ubiquitous genes identified by Koonin [14]. The bacteria families, genera and species analyzed in this study are summarized in **Table 1**. Only one gene sequence per taxon was retained in cases where the isolates shared 100% gene sequence similarity to avoid bias due to primer choice for a taxon. Sequencing of genomes from representatives of about 200 other high G+C content bacteria are currently in progress (http://www.genomesonline.org). The intraspecies similarities were performed *in silico* on 53 strains belonging to 14 species of the subclass Actinobacteridae and experimentally on 20 clinical isolates of *Mycobacterium abscessus* (**Table 3**).

### Common set of gene database for primers designation

A common set of gene related sequences were retrieved from the available whole-genome sequences. This was facilitated with BLAST searches (http://www.ncbi.nlm.nih.gov/BLAST/genome) performed against the 50 Actinobacteria genome sequences available at the NCBI website (http://www.ncbi.nlm.nih.gov/sutils/genom_table.cgi/) using the orthologues of a common set of genes derived from *Mycobacterium tuberculosis* (GenBank accession number CP000611) and *Propionibacterium acnes* (GenBank accession number AE017283) as queries (**Table 1**). The common set of genes thus obtained were aligned using clustal X program, version 1.83, in the PHYLIP software package [62]. New broad range primers were designed for amplification and sequencing using SVARAP software which analyzes and graphically represents the variability in stretches of 50-bp along the nucleotide sequence [40], [41]. A BLAST search was performed to check potential primers for unspecific amplification. To ascertain primer efficacy and amplification efficiency (**Table 2**), a panel of genera of Actinobacteria was tested (**Table 3**). 16S rRNA amplification with primer pair fD1-rP2 [63] was used to ensure DNA extraction.

### Individual gene fragment and whole-genome conserved region alignments

After alignment with clustal X, the sequence similarities of the individual gene fragment were determined using BioEdit v7.0.9 (Ibis Biosciences, Carlsbad, CA). To determine the genome conserved sequence similarity, pairs of whole genomes were aligned by using the MUMmer application [64] with the following parameters: breaken = 500, minCluster = 40, diagFactor = 0.15, maxGap = 250 and minMatch = 12. To ensure that all possible alignments were found, the reference and query files were swapped and each pair was reanalyzed. When two neighbouring sequence regions shared overlapping endpoints, the common segment was divided equally between them. Two similarity estimates were calculated from each genomic sequence comparison. DNA sequence similarity for conserved regions was calculated as the mean sequence identity of the homologous regions, weighted by each region's length in nucleotides.

### Amplification and sequencing methods

PCR was conducted in 50 µl volumes with 25 µl of hotstart master mix and 20 µl water (Qiagen, Germantown, MD) with 15 min at 95°C followed by 35 cycles of 95°C for 1 min, 50°C, 52°C and 60°C depending on the primers (**Table 2**) for 1.30 min and 72°C for 1 min with a final extension step at 72°C for 10 min. Sequence reactions utilized the ABI Prism Big Dye v3.1 terminator cycle sequencing ready reaction kit (Perkin Elmer Applied Biosystems, Foster City, Calif) using the following program: an initial denaturation step of 1 min at 96°C followed by 25 cycles of denaturation at 96°C for 10 s, annealing at 50°C for **5 **s and elongation at 60°C for 4 min. Products of sequencing reactions were recorded with ABI Prism 3130*xl* sequencer following the protocol of the supplier (Perkin Elmer Applied Biosystems).

### Phylogenetic analysis

After the genomic statistical analysis, the sequences in GenBank were re-examined to increase the number of strains incorporated in the phylogenetic analysis. Phylogenetic trees were constructed from the common set of gene sequences using the neighour-joining method with Kimura's two parameter distance correction model with 1,000 bootstrap replications in MEGA version 4.0 software package [65]. The primer binding sites were eliminated from the sequences prior to computer analysis.

### Statistical analysis

The chi-square test is used to compare the bootstrap values at the nodes of the phylogenetic trees. A p-value of <0.05 was considered significant.

## Supporting Information

Table S1 Family and species analyzed within the phylum Actinobacteria.
(0.12 MB DOC)

## References

[1] Stackebrandt E, Frederiksen W, Garrity GM, Grimont PA, Kämpfer P (2002). Report of the ad hoc committee for the re-evaluation of the species definition in bacteriology.. Int J Syst Evol Microbiol.

[2] Wertz JE, Goldstone C, Gordon DM Riley MA (2003). A molecular phylogeny of enteric bacteria and implications for a bacterial species concept.. J Evol Biol.

[3] Zeigler DR (2003). Gene sequences useful for predicting relatedness of whole genomes in bacteria.. Int J Syst Evol Microbiol.

[4] Adekambi T, Drancourt M (2004). Dissection of phylogenetic relationships among 19 rapidly growing *Mycobacterium* species by 16S rRNA, *hsp*65, *sod*A, *rec*A and *rpo*B gene sequencing.. Int J Syst Evol Microbiol.

[5] Devulder G, Pérouse de Montclos M, Flandrois JP (2005). A multigene approach to phylogenetic analysis using the genus *Mycobacterium* as a model.. Int J Syst Evol Microbiol.

[6] Mignard S, Flandrois JP (2008). A seven-gene, multilocus, genus-wide approach to the phylogeny of mycobacteria using super trees.. Int J Syst Evol Microbiol.

[7] Adekambi T, Raoult D, Drancourt M (2006). *Mycobacterium barrassiae* sp. nov., a *Mycobacterium moriokaense* group species associated with chronic pneumonia.. J Clin Microbiol.

[8] Stinear TP, Seemann T, Harrison PF, Jenkin GA, Davies JK (2008). Insights from the complete genome sequence of *Mycobacterium marinum* on the evolution of *Mycobacterium tuberculosis*.. Genome Res.

[9] Coenye T, Gevers D, Van de Peer Y, Vandamme P, Swings J (2005). Towards a prokaryotic genomic taxonomy.. FEMS Microbiol Rev.

[10] Gevers D, Cohan FM, Lawrence JG, Spratt BG, Coenye T (2005). Opinion: Re-evaluating prokaryotic species.. Nat Rev Microbiol.

[11] Yamamoto S, Harayama S (1996). Phylogenetic analysis of *Acinetobacter* strains based on the nucleotide sequences of *gyr*B genes and on the amino acid sequences of their products.. Int J Syst Bacteriol.

[12] Gil R, Silva FJ, Peretó J, Moya A (2004). Determination of the core of a minimal bacterial gene set. Microbiol.. Mol Biol *Rev*.

[13] Santos SR, Ochman H (2004). Identification and phylogenetic sorting of bacterial lineages with universally conserved genes and proteins.. Environ Microbiol.

[14] Koonin EV (2003). Comparative genomics, minimal gene-sets and the last universal common ancestor.. Nat Rev Microbiol.

[15] Koonin EV (2000). How many genes can make a cell: the minimal-gene-set concept.. Annu Rev Genomics Hum Genet.

[16] Mushegian AR, Koonin EV (1996). A minimal gene set for cellular life derived by comparison of complete bacterial genomes.. Proc Natl Acad Sci USA.

[17] Pallen MJ, Wren BW (2007). Bacterial pathogenomics.. Nature.

[18] Zhi XY, Li WJ, Stackebrandt E (2009). An update of the structure and 16S rRNA gene sequence-based definition of higher ranks of the class Actinobacteria, with the proposal of two new suborders and four new families and emended descriptions of the existing higher taxa.. Int J Syst Evol Microbiol.

[19] Atlas RM (1997). Principles of Microbiology..

[20] Embley TM, Stackebrandt E (1994). The molecular phylogeny and systematics of the actinomycetes.. Annu Rev Microbiol.

[21] Stackebrandt E, Rainey FA, Ward-Rainey NL (1997). Proposal for a new hierarchic classification system *Actinobacteria classis* nov.. Int J Syst Bacteriol.

[22] Ventura M, Canchaya C, Del Casale A, Dellaglio F, Neviani E (2006). Analysis of bifidobacterial evolution using a multilocus approach.. Int J Syst Evol Microbiol.

[23] Gao B, Gupta RS (2005). Conserved indels in protein sequences that are characteristic of the phylum Actinobacteria.. Int J Syst Evol Microbiol.

[24] Roller C, Ludwig W, Schleifer KH (1992). Gram-positive bacteria with a high DNA G+C content are characterized by a common insertion within their 23S rRNA genes.. J Gen Microbiol.

[25] Gao B, Paramanathan R, Gupta RS (2006). Signature proteins that are distinctive characteristics of Actinobacteria and their subgroups.. Antonie Van Leeuwenhoek.

[26] Ara I, Bakir MA Kudo T (2008). Transfer of *Catellatospora koreensis* Lee, et al. 2000 as *Catelliglobosispora koreensis* gen. nov., comb. nov. and *Catellatospora tsunoense* Asano et al. 1989 as *Hamadaea tsunoensis* gen. nov., comb. nov., and emended description of the genus *Catellatospora* Asano and Kawamoto 1986 emend. Lee and Hah 2002.. Int J Syst Evol Microbiol.

[27] Chun J, Blackall LL, Kang SO, Hah YC, Goodfellow M (1997). A proposal to reclassify *Nocardia pinensis* Blackall et al. as *Skermania piniformis* gen. nov., comb. nov.. Int J Syst Bacteriol.

[28] Kämpfer P, Andersson MA, Rainey FA, Kroppenstedt RM, Salkinoja-Salonen M (1999). *Williamsia muralis* gen. nov., sp. nov., isolated from the indoor environment of a children's day care centre.. Int J Syst Bacteriol.

[29] Lee SD (2006). *Blastococcus jejuensis* sp. nov., an actinomycete from beach sediment, and emended description of the genus *Blastococcus* Ahrens and Moll 1970.. Int J Syst Evol Microbiol.

[30] Lehnen A, Busse HJ, Frölich K, Krasinska M, Kämpfer P (2006). *Arcanobacterium bialowiezense* sp. nov. and *Arcanobacterium bonasi* sp. nov., isolated from the prepuce of European bison bulls (Bison bonasus) suffering from balanoposthitis, and emended description of the genus *Arcanobacterium* Collins et al. 1983.. Int J Syst Evol Microbiol.

[31] McKenzie CM, Seviour EM, Schumann P, Maszenan AM, Liu JR (2006). Isolates of ‘Candidatus *Nostocoida limicola*’ Blackall et al. 2000 should be described as three novel species of the genus *Tetrasphaera*, as *Tetrasphaera jenkinsii* sp. nov., *Tetrasphaera vanveenii* sp. nov. and *Tetrasphaera veronensis* sp. nov.. Int J Syst Evol Microbiol.

[32] Rainey FA, Klatte S, Kroppenstedt RM, Stackebrandt E (1995). *Dietzia*, a new genus including *Dietzia maris* comb. nov., formerly *Rhodococcus maris*.. Int J Syst Bacteriol.

[33] Soddell JA, Stainsby FM, Eales KL, Kroppenstedt RM, Seviour RJ (2006). *Millisia brevis* gen. nov., sp. nov., an actinomycete isolated from activated sludge foam.. Int J Syst Evol Microbiol.

[34] Butler WR, Floyd MM, Brown JM, Toney SR, Daneshvar MI (2005). Novel mycolic acid-containing bacteria in the family *Segniliparaceae* fam. nov., including the genus *Segniliparus* gen. nov., with descriptions of *Segniliparus rotundus* sp. nov. and *Segniliparus rugosus* sp. nov.. Int J Syst Evol Microbiol.

[35] Gogarten JP, Doolittle WF, Lawrence JG (2002). Prokaryotic evolution in light of gene transfer.. Mol Biol Evol.

[36] Gogarten JP, Townsend J (2005). Horizontal gene transfer, genome innovation and evolution.. Nat Rev Microbiol.

[37] Koonin EV, Makarova KS, Aravind L (2001). Horizontal gene transfer in prokaryotes: quantification and classification.. Annu Rev Microbiol.

[38] Novichkov PS, Omelchenko MV, Gelfand MS, Mironov AA, Wolf YI (2004). Genome-wide molecular clock and horizontal gene transfer in bacterial evolution.. J Bacteriol.

[39] Stach JE, Maldonado LA, Ward AC, Goodfellow M, Bull AT (2003). New primers for the class Actinobacteria: application to marine and terrestrial environments.. Environ Microbiol.

[40] Adekambi T, Colson P Drancourt M (2003). *rpo*B-based identification of nonpigmented and late-pigmenting rapidly growing mycobacteria.. J Clin Microbiol.

[41] Colson P, Tamalet C, Raoult D (2006). SVARAP and aSVARAP: simple tools for quantitative analysis of nucleotide and amino acid variability and primer selection for clinical microbiology.. BMC Microbiol.

[42] Rogall T, Wolters J, Flohr T, Böttger EC (1990). Towards a phylogeny and definition of species at the molecular level within the genus *Mycobacterium*.. Int J Syst Bacteriol.

[43] Ninet B, Monod M, Emler S, Pawlowski J, Metral C (1996). Two different 16S rRNA genes in a mycobacterial strain.. J Clin Microbiol.

[44] Ueda K, Seki T, Kudo T, Yoshida T, Kataoka M (1999). Two distinct mechanisms cause heterogeneity of 16S rRNA.. J Bacteriol.

[45] Acinas SG, Marcelino LA, Klepac-Ceraj V, Polz MF (2004). Divergence and redundancy of 16S rRNA sequences in genomes with multiple rrn operons.. J Bacteriol.

[46] Boucher Y, Douady CJ, Sharma AK, Kamekura M, Doolittle WF (2004). Intragenomic heterogeneity and intergenomic recombination among haloarchaeal rRNA genes.. J Bacteriol.

[47] Wang Y, Zhang Z (2000). Comparative sequence analyses reveal frequent occurrence of short segments containing an abnormally high number of non-random base variations in bacterial rRNA genes.. Microbiology.

[48] Yap WH, Zhang Z, Wang Y (1999). Distinct types of rRNA operons exist in the genome of the actinomycete *Thermomonospora chromogena* and evidence for horizontal transfer of an entire rRNA operon.. J Bacteriol.

[49] Ventura M, Canchaya C, Tauch A, Chandra G, Fitzgerald GF (2007). Genomics of Actinobacteria: tracing the evolutionary history of an ancient phylum.. Microbiol Mol Biol Rev.

[50] Richert K, Brambilla E Stackebrandt E (2007). The phylogenetic significance of peptidoglycan types: Molecular analysis of the genera *Microbacterium* and *Aureobacterium* based upon sequence comparison of *gyr*B, *rpo*B, *rec*A and *ppk* and 16SrRNA genes.. Syst Appl Microbiol.

[51] Stackebrandt E, Brambilla E Richert K (2007). Gene sequence phylogenies of the family microbacteriaceae.. Curr Microbiol.

[52] Guo Y, Zheng W, Rong X, Huang Y (2008). A multilocus phylogeny of the *Streptomyces griseus* 16S rRNA gene clade: use of multilocus sequence analysis for streptomycete systematics.. Int J Syst Evol Microbiol.

[53] Adekambi T, Drancourt M, Raoult D (2009). *rpo*B gene as a tool for clinical microbiologist.. Trends Microbiol.

[54] Jordan IK, Rogozin IB, Wolf YI Koonin EV (2002). Essential genes are more evolutionarily conserved than are nonessential genes in bacteria.. Genome Res.

[55] Gerdes SY, Scholle MD, Campbell JW, Balázsi G, Ravasz E (2003). Experimental determination and system level analysis of essential genes in *Escherichia coli* MG1655.. J Bacteriol.

[56] Kobayashi K, Ehrlich SD, Albertini A, Amati G, Andersen KK (2003). Essential *Bacillus subtilis* genes.. Proc Natl Acad Sci USA.

[57] Edwards SV (2009). Is a new and general theory of molecular systematics emerging?. Evolution.

[58] Adekambi T, Shinnick TM, Raoult D, Drancourt M (2008). Complete *rpo*B gene sequencing as a suitable supplement to DNA-DNA hybridization for bacterial species and genus delineation.. Int J Syst Evol Microbiol.

[59] Konstantinidis KT, Tiedje JM (2005). Genomic insights that advance the species definition for prokaryotes.. Proc Natl Acad Sci USA.

[60] Bininda-Emonds OR (2004). The evolution of supertrees.. Trends Ecol Evol.

[61] Garrity GM, Bell JA, Lilburn, TG (2004). Bergey's Manual of Systematic Bacteriology.2nd..

[62] Thompson JD, Gibson TJ, Plewniak F, Jeanmougin F, Higgins DG (1997). The CLUSTAL_X windows interface: flexible strategies for multiple sequence alignment aided by quality analysis tools.. Nucleic Acids Res.

[63] Weisburg WG, Barns SM, Pelletier DA, Lane DJ (1991). 16S ribosomal DNA amplification for phylogenetic study.. J Bacteriol.

[64] Delcher AL, Phillippy A, Carlton J, Salzberg SL (2002). Fast algorithms for large-scale genome alignment and comparison.. Nucleic Acids Res.

[65] Tamura K, Dudley J, Nei M, Kumar S (2007). MEGA4: Molecular Evolutionary Genetics Analysis (MEGA) software version 4.0.. Mol Biol Evol.

