# Supplementary material for: Core Gene Set As the Basis of Multilocus Sequence Analysis of the Subclass Actinobacteridae
Source: PLoS One. 2011 Mar 31;6(3):e14792. doi: 10.1371/journal.pone.0014792 (PMC3069002; doi:10.1371/journal.pone.0014792)
Supplement: Table S1 — Family and species analyzed within the phylum Actinobacteria. (0.12 MB DOC) [file pone.0014792.s001.doc]

Table S1. Family and species analyzed within the phylum Actinobacteria

| Family | Species | GenBank accession number |
| --- | --- | --- |
| *Corynebacteriaceae* | *Corynebacterium diphteriae* NCTC 13129 | BX248358 |
|  | *Corynebacterium efficiens* YS-314 | BA000035 |
|  | *Corynebacterium glutamicum* ATCC 13032 | BA000036 |
|  | *Corynebacterium glutamicum* R | AP009044 |
|  | *Corynebacterium jeikeium* K411 | CR931997 |
|  | *Corynebacterium urealyticum* DSM 7109 | AM942444 |
| *Mycobacteriaceae* | *Mycobacterium leprae* TN | AL583922 |
|  | *Mycobacterium bovis* AF2122/97 | BX248343 |
|  | *Mycobacterium bovis* BCG Pasteur 1173P2 | AM408590 |
|  | *Mycobacterium tuberculosis* CDC1551 | AE000516 |
|  | *Mycobacterium tuberculosis* H37Rv | BX842581 |
|  | *Mycobacterium tuberculosis* H37Ra | CP000611 |
|  | *Mycobacterium tuberculosis* F11 | CP000717 |
|  | *Mycobacterium avium* 104 | CP000479 |
|  | *Mycobacterium paratuberculosis* K-10 | AE016958 |
|  | *Mycobacterium ulcerans* Agy99 | CP000325 |
|  | *Mycobacterium marinum* M | CP000854 |
|  | *Mycobacterium smegmatis* MC^2^ 155 | CP000480 |
|  | *Mycobacterium monacense* MCS | CP000384 |
|  | *Mycobacterium monacense* KMS | CP000518 |
|  | *Mycobacterium monacense* JLS | CP000580 |
|  | *Mycobacterium abscessus* CI P104536T | CU458896 |
|  | *Mycobacterium gilvum* PYR-GCK | CP000656 |
|  | *Mycobacterium vanbaalenii* PYR-1 | CP000511 |
| *Nocardiaceae* | *Nocardia farcinica* IFM 10152 | AP006618 |
|  | *Rhodococcus* sp. RHA1 | CP000431 |
| *Acidothermaceae* | *Acidothermus cellulolyticus* 11B | CP000481 |
| *Frankiaceae* | *Frankia alni* ACN14a | CT573213 |
|  | *Frankia* sp. Ccl3 | CP000249 |
|  | *Frankia* sp. EAN1pec | CP000820 |
| *Kineosporiaceae* | *Kineococcus radiotolerans* SRS30216 | CP000750 |
| *Microbacteriaceae* | *Leifsonia xyli subsp. xyli str.* CTCB07 | AE016822 |
|  | *Clavibacter michiganensis* subsp. *michiganensis* NCPPB 382 | AM711867 |
|  | *Clavibacter michiganensis subsp. sepedonicus* ATCC 33113 | AM849034 |
| *Micrococcaceae* | *Arthrobacter aurescens* TC1 | CP000474 |
|  | *Arthobacter* sp. FB24 | CP000454 |
|  | *Renibacterium salmoninarum* ATCC 33209 | CP000910 |
|  | *Kocuria rhizophila* DC2201 | AP009152 |
| *Nocardioidaceae* | *Nocardiodes* sp. JS614 | CP000509 |
| *Propionibacteriaceae* | *Propionibacterium acnes* KPA171202 | AE016822 |
| *Pseudonocardiaceae* | *Saccharopolyspora erythraea* NRRL 2338 | AM420293 |
| *Streptomycetaceae* | *Streptomyces avermitilis* MA-4680 | BA000030 |
|  | *Streptomyces coelicolor* A3(2) | AL939124 |
|  | *Streptomyces griseus* subsp. *griseus* NBRC 13350 | AP009493 |
| *Nocardiopsaceae* | *Thermobifida fusca* YX | CP000088 |
| *Bifidobactereriaceae* | *Bifidibavterium adolescentis* ATCC 15703 | AP009256 |
|  | *Bifidibavterium longum* DJO10A | CP000605 |
|  | *Bifidibavterium longum* NCC2705 | AE014295 |
| *Micromonosporaceae* | *Salinispora arenicola* CNS205 | CP000850 |
|  | *Salinispora tropica* CNB-440 | CP000667 |

Taxonomy from Bergey’s Manual of Systematic Bacteriology [61]
